# Multiple overlapping microwave ablation in benign thyroid nodule: a single-center 24-month study

**DOI:** 10.1530/ETJ-22-0175

**Published:** 2023-01-25

**Authors:** Eduardo Crespo Vallejo, Antonio Hermosin, Manuel Gargallo, Álvaro Villalba, Eduardo Daguer, José Flores, Javier Periañez, Joaquim Amorín, Ernesto Santos

**Affiliations:** 1 Interventional Radiology, Hospital Universitario Fundacion Jiménez Diaz, Madrid, Spain; 2 Endocrinology, Hospital Universitario Fundacion Jiménez Diaz, Madrid, Spain; 3 Interventional Radiology, Memorial Sloan Kettering Cancer Center, New York, NY, USA

**Keywords:** microwave, thyroid nodule, multiple overlapping ablation, radiofrequency, moving shot technique

## Abstract

**Objective:**

This study aimed to evaluate the safety and long-term efficacy using the multiple overlapping ablation technique with a novel non-cooled microwave system in benign symptomatic thyroid nodules.

**Methods:**

This prospective cohort single-center study collected complication data from the start of the procedure to 30 days postoperatively and evaluated the safety and effectiveness with a follow-up of 24 months. Ultrasound examinations were performed to determine the volume shrinkage during follow-up. Thyroid function cosmetic and symptoms scores and satisfaction degree were evaluated.

**Results:**

A total of 30 symptomatic benign thyroid nodules were treated by microwave ablation using a power between 15 and 30 W depending on the size of the nodule to be treated. The volume reduction rates in months 1, 3, 6, 9, 12, and 24 after ablation were 32, 59, 67, 69, 73, and 81%, respectively. The mean symptom score and mean cosmetic score before treatment were 4 and 3, respectively, while after treatment they dropped to 3 and 1, respectively. Thyroid function indicators fluctuated in the normal range and those with hyperthyroidism recovered to normal parameters. One case of temporary laryngeal paralysis occurred postoperatively and fully recovered in less than 3 months.

**Conclusions:**

The novel microwave ablation system presented herein can help achieve good clinical success rate in benign thyroid nodules with a satisfying safety profile. The microwave ablation performed with the multiple overlapping ablation technique could be a good alternative to surgery and radiofrequency ablation in the management of benign thyroid nodules.

## Introduction

The prevalence of thyroid nodules (TNs) in the general population is approximately 60% (1, 2). Most TNs are benign and do not require any kind of treatment. For large nodules causing compressive symptoms, hormonal dysfunction and/or esthetic discomfort (3, 4) surgery is traditionally viewed as the first-line treatment. However, surgery for benign TNs is likely to be presently overused in Europe (5), and ultrasound-guided interventional techniques stand as a reasonable alternative treatment to surgery, considering their lower costs and the lower rate of reported complications.

In the management of benign TN, clinicians often face the dilemma of whether to maintain follow-up for years or send the patient to surgery. Thyroid surgery requires general anesthesia, and hospitalization usually takes several days. In addition to the permanent postoperative surgical hypothyroidism, also likely in the case of conservative procedures (e.g. lobo-isthmectomy), other complications, such as hypoparathyroidism and recurrent laryngeal nerve injury, may occur (6). Moreover, patients will have a permanent scar on the neck. Bernardi *et al.* recently published a comparative cost analysis which concluded that radiofrequency ablation (RFA) causes fewer complications and is more economical compared to surgery (7).

Given the above context, clinicians are compelled to incorporate thermoablation as a preferred therapeutic option in the management of benign TNs. The 2020 European Thyroid Association Clinical Practice Guideline for the Use of Image-Guided Ablation in Benign Thyroid Nodules (3) has clearly stated that image-guided thermal ablation (TA) should be considered as a cost- and risk-effective alternative to surgical treatment or observation alone in adult patients who have benign TNs that cause pressure symptoms and/or cosmetic concerns and decline surgery. RFA is the most utilized system, but microwave ablation (MWA) is an interesting new option with the potential to overcome some limitations of RFA (8, 9, 10, 11).

Studies have compared RFA to MWA, and in most of them, RFA presents better results than MWA. However, none of these studies have used microwave antennas specifically developed for the ablation of TNs (12, 13, 14).

In this study, we conducted a safety (from the start of procedure up to 30 days postoperatively) and efficacy analysis (up to 24 months of follow-up) of our initial experience in ablating benign TNs (BTN) using a novel MWA system with non-cooled 17 or 18 G antennas and multiple overlapping ablation (MOA) technique.

## Material and methods

### Study design and patient selection

This prospective cohort single-center study was performed in accordance with the ethical standards prescribed by the Declaration of Helsinki. Additionally, this study was approved by the Fundación Jiménez Díaz Clinical Research Ethics Committee, and all patients gave their informed consent for participation in the research study. Written consent has been obtained from each patient after full explanation about the benefits and possible adverse effects of the procedure before participating in this study. Treatment was performed according to standard practice, and data were prospectively collected from January 2019 to March 2021 in our institution.

All patients presented ultrasound (US) findings suggestive of BTN and had benign cytologic confirmation by two consecutive US‐guided fine‐needle aspiration cytology (FNAC) procedures. The patients were selected based on the following inclusion criteria: (i) anxiety about TNs and willingness to undergo treatment, (ii) presence of either compressive and/or cosmetic problems, (iii) refusal to undergo surgery or has contraindications to surgery, and (iv) subclinical or symptomatic hyperthyroidism. Patients with the following were excluded: (i) coagulation dysfunction, (ii) retrosternal growth, and (iii) pregnant. The size of the nodule to be ablated was never an exclusion criterion if the entire nodule was visible in US.

Before the procedure, conventional clinical examination, US, two US-guided FNACs performed separately, and laboratory tests were performed. A complete visualization of the thyroid nodule in diagnostic US study is necessary to consider the possibility of thermal ablation. At registration, patients were evaluated for the compression symptoms using a 10-cm visual analog scale (compressive score, 0–10). The endocrinologist uses the following cosmetic grading system: 1, no palpable mass; 2, a palpable mass but no cosmetic problem; 3, cosmetic problem on swallowing only; and 4, readily detected cosmetic problem (aesthetic score, 1–4). During US, nodules were evaluated for their suitability for thermal ablation including position, size, volume, solid/cystic proportions, echogenicity, and volume. The volume of each nodule was calculated before MWA according to the ellipsoid formula: V=1⁄4 πabc/6 (where V is the volume; a, largest diameter; and b and c, the two other orthogonal diameters). Regarding basal hormonal analytics, thyroid-stimulating hormone (TSH) and free thyroxine (FT4) levels were exclusively obtained.

### Ablation procedure

All interventions were performed by an interventional radiology team, consisting of four physicians with >3 years of experience in thyroid ablation. The anesthesiologist was responsible for sedation and patient’s well-being during the procedure.

AplioTM 500 US machine (Toshiba Medical Systems) was used for MWA treatment guidance. A novel non-cooled MW system (TATO, Biomedical, Florence, Italy) was used for the ablative treatment. The microwave antenna was 17 G or 18 G depending on the volume of the nodule, usually using the 17-G needle for nodules with a maximum diameter of >2 cm.

To ensure the safety of the procedure, patients fasted for at least 8 h and a venous catheter was inserted in a forearm vein before the procedure. Although the procedure is usually performed exclusively with local anesthesia, when the patient was anxious, afraid of the procedure, or had a history of syncope during minor interventions such as blood collection, the anesthesiologist was asked to provide minimal sedation and/or anxiolysis with midazolam and/or fentanyl, preserving in any case patient’s consciousness to talk to him/her during the whole procedure.

The patient was in the supine position with a small pillow under the neck to expose the cervical region sufficiently. A multiparameter monitor continuously recorded the patient’s vital signs such as blood pressure, partial pressure of oxygen, pulse rate, and ECG during the procedure. All procedures were completed under a sterile environment and local anesthesia with 1% lidocaine. With the appropriate pressure of the US probe, the target nodule and its adjacent structures were clearly identified in real-time US, and preprocedure planning including the best approach to the target nodule was confirmed. In most of the ablations, we selected a trans-isthmic approach; only when this route was impossible, a direct puncture of the nodule was employed.

**Figure 1:**
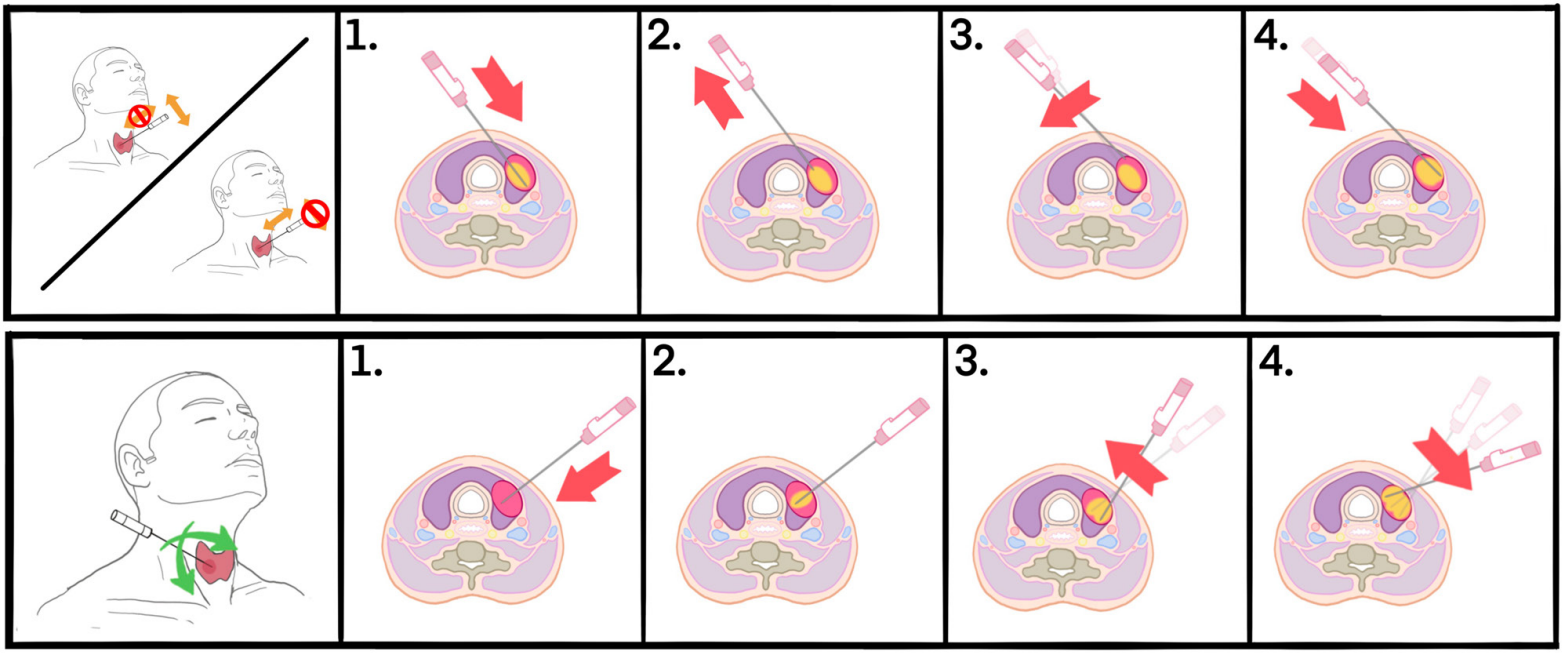
In this graph, two types of multiple overlapping ablation techniques used with the MW TATO system are shown. The first line presents the classic trans-isthmic access with partial withdrawal of the needle once the ablation has stopped, to go to the next ablative area once the first one has been achieved. In the second line, with a direct access to the nodule, the needle was not withdrawn so much to go to the next selected area; therefore, the ablation was always kept active.

For all procedures, we utilized a MWA system (TATO, Biomedical) that does not require internal cooling, allowing for specific very thin and small antennas to be utilized (17 G and 18 G, 10 cm and 8 cm in length). MWA allows for use of the so-called MOA that requires less repositioning of the antenna in comparison with the MST commonly used for RFA. MOA with MWA should lead to faster procedural times even in case of large nodules (>50 mL). In cases in which access is gained through the isthmus, the classic technique is used (Fig. 1). We begin the ablation in the deepest and most caudal parts of the nodule and then withdraw the needle almost completely, keeping the tip inside the nodule to redirect it immediately toward the more superficial and cranial areas once ablation was achieved in the first area. Using the selected antenna, it is quite common to determine when an ablative area has been achieved because we will see echographic changes consisting of a hypoechoic ovoidal morphology area around the needle tip that at some point does not progress over time. In nodules with cystic or spongiform component, greater amounts of steam generated usually dissipate quickly and allow visualization of the needle in virtually the entire procedure.

When the isthmus is too small or does not allow easy access to the nodule because of its location, we use direct puncture of the nodule. In these cases, we also try to penetrate the nodule at its equator to initially target the most caudal part. With this access, mobility within the nodule is less limited by the patient’s anatomy, such as the bone structures; therefore, we move the needle inside the nodule, also in a cranial direction, without retrieving or stopping the ablation. With this technique, we select a lower power, usually 15 W, and in this way, we are moving and destroying the nodule inside at all times. A thermomechanical destructive effect is achieved. Cervelli *et al.* described in 2017 the benefits of a direct access to the nodule, which consist basically better maneuverability within the nodule that requires less antenna retractions and better US control (15).

**Figure 2:**
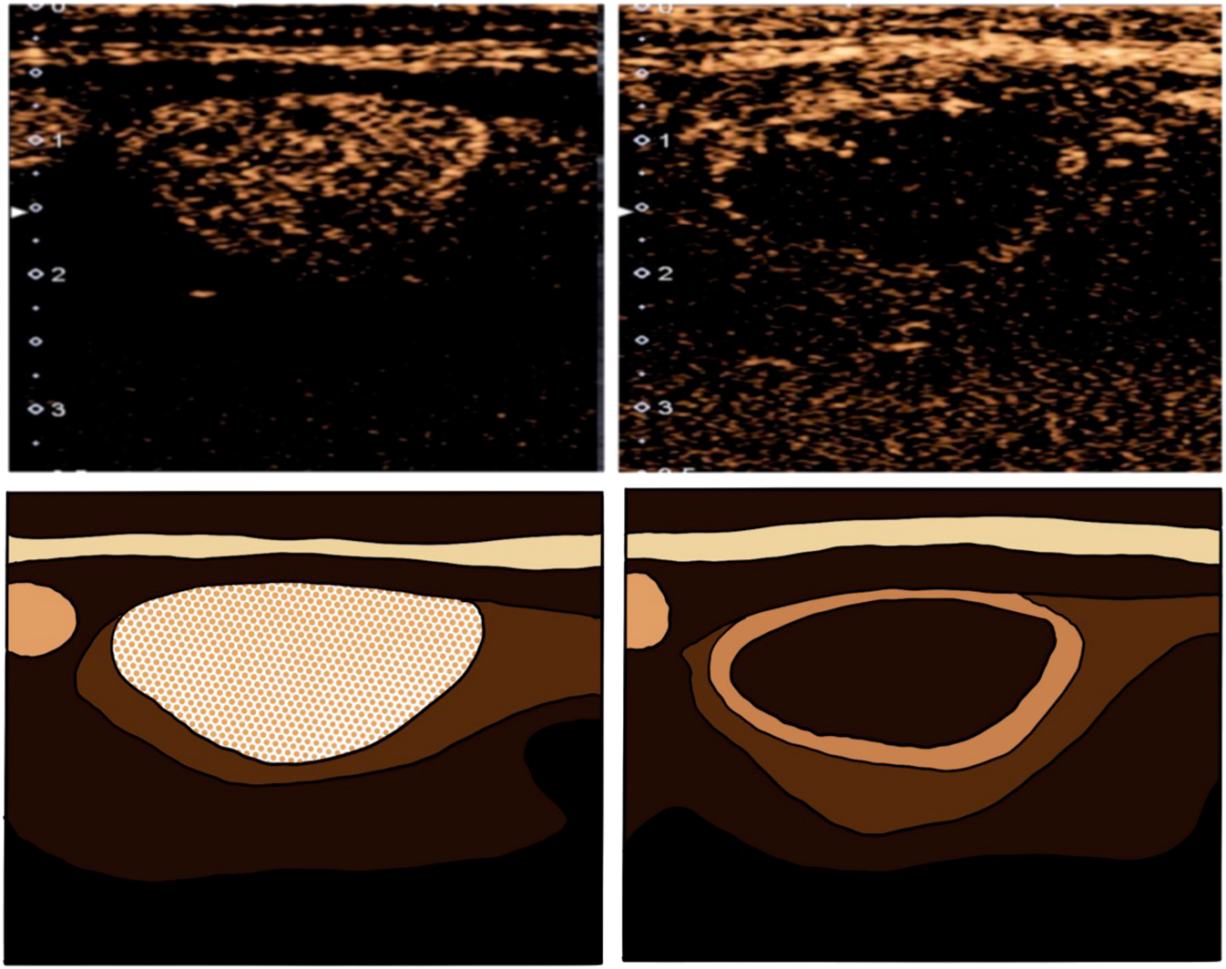
Use of ultrasound contrast before and immediately after ablation ensures treatment of at least 80% of the nodule.

In those thyroid nodules that showed significant flow in the initial doppler ultrasound study, contrast-enhanced US (CEUS) was used before and immediately after the procedure to ensure sufficient ablation. Sulfur hexafluoride (SonoVue, Bracco, Milan, Italy) was used as ultrasound contrast agent and CEUS was performed after bolus injection of SonoVue (2.4 mL), followed by a 5 mL of normal saline flush. If the final test did not demonstrate devascularization with an unenhanced pattern in >80% of the nodule, the treatment is continued until this rate is safely achieved (Fig. 2).

US examination allowed monitoring of complications during and after the procedure. Ice was applied to the patient’s neck at the end of the procedure to prevent edema of the superficial tissue. Pain killers were not administered during the procedure or at the end of the procedure if not were requested by the patient.

### Follow-up of the patients

Follow-up was performed at months 1, 3, 6, 12, and 24 after the treatment, including consultation and US. Thyroid function tests (TSH and FT4), symptom score, and cosmetic score were evaluated during and at the end of the follow-up.

The volume reduction rate (VRR) of the treated nodule was calculated based on the following formula: VRR (baseline volume − post-treatment volume) / baseline volume × 100%.

### Efficacy and safety outcomes

Technical success was ≥50% reduction in the nodule volume, and clinical success was defined as improvement of aesthetics and compression symptoms.

Safety outcome (complications and side effects) followed that reported by the international working group on image-guided tumor ablation. Major complications include substantial morbidity and disability that increases the level of care, hospital admission, hemorrhage requiring blood transfusion, and permanent voice change. Other complications were identified as minor complications, such as pain, transient voice change, vomiting, and skin burns.

### Statistical analysis

Demographic information included age and sex, characteristics of the nodule to be treated, treatment indications, thickness of the electrode and power, changes in nodule size during follow-up, complications, post-procedure pain, symptoms score before and after the procedure, cosmetic score before and after the procedure, and degree of overall satisfaction 12 months after the procedure.

The statistical analysis was conducted using the RStudio software application (version 1.3.1093). Descriptive statistical analysis was performed, and results are presented as frequency, total, and proportion represented by each categorical variable. For numerical variables, the mean and s.d. were shown for variables that are normally distributed, along with the median and 0.25 and 0.75 quartile values.

The inferential statistical analysis was performed in each case and for each of the variables, the mean in each group and s.d.. The *P*-value resulting from the comparison of the groups was also included through Student’s *t*-test when two groups were compared. To establish differences between the volumes, the means at different times were analyzed using the analysis of the variance test. These tests were performed for normally distributed variables. If the tests were not given, the nonparametric Mann–Whitney *U* or Kruskal–Wallis tests are applied, as appropriate.

## Results

From January 2019 to March 2021, 35 patients who had undergone MWA of TNs were enrolled in this study. Following treatment, five patients were lost before the 12-month follow-up because of the COVID-19 pandemic. A final sample of 30 patients with 30 nodules was followed for 24 months (Table 1).

**Table 1:** Demographics, nodule features, indication for treatment, and size evolution during the follow-up after the microwave ablation.

Patient no.	Age/sex	Composition^a^	Gammagraphy	Baseline nodule		Indication^b^	Probe	Power	Volume evolution in follow-up (mL)				VRR
				Diameter (mm)	Volume (mL)				3 M	6 M	12 M	24 M	
1	54/F	S	Cold	24 × 42 × 56	29.55	AE,C	17 G	25 W	9.55	3.38	2.58	2.32	92.12
2	53/F	M	Hot	19 × 19 × 23	4.34	H	17 G	20 W	1	0.47	0.36	0.3	93.06
3	50/F	S	Cold	21 × 28 × 39	12	AE	17 G	25 W	1.72	0.98	0.75	0.71	94
4	51/F	S	Cold	24 × 38 × 60	28.65	H	17 G	20 W	13.13	10.02	8.24	6.75	76.41
5	54/M	E	Cold	17 × 20 × 25	4.45	G	17 G	25 W	1.39	1.49	1.17	0.85	80.72
6	51/F	M	Cold	30 × 30 × 50	23.56	AE,C	17 G	20 W	6.35	3.12	2.19	1.79	92.37
7	46/F	S	Cold	25 × 27 × 44	15.55	C,AE	18 G	15 W	10.07	6.9	4.94	4.05	73.89
8	78/F	S	Hot	13 × 20 × 21	2.85	H,AE	17 G	25 W	0.91	0.85	0.67	0.57	80.05
9	59/F	E	Hot	14 × 17 × 30	3.73	C,H	17 G	20 W	1.19	0.75	0.71	0.59	84.11
10	40/F	S	Cold	20 × 25 × 33	8.63	C,AE,G	17 G	20 W	3.73	2.69	2.45	2.02	7.65
11	57/F	S	Cold	28 × 32 × 44	20.64	AE	18 G	15 W	6.01	4.9	4.22	3.11	84.93
12	48/F	M	Hot	20 × 22 × 28	6.45	H	17 G	20 W	3.84	3.2	2.89	2.19	6.59
13	45/F	S	Cold	23 × 35 × 40	16.85	AE	17 G	25 W	6.97	3.67	2.76	2.38	85.85
14	35/F	S	Cold	20 × 26 × 39	10.61	AE	17 G	25 W	3.61	2.66	1.96	1.38	85.68
15	54/F	S	Cold	27 × 37 × 40	20.92	AE	18 G	15 W	8.92	8.48	6.33	5.32	74.55
16	50/F	S	Cold	15 × 21 × 35	5.77	AE,C	18 G	15 W	3.87	2.2	1.52	0.84	8.53
17	50/F	S	Cold	35 × 35 × 60	38.48	AE	18 G	15 W	24.19	16.17	16.57	17.08	55.59
18	47/F	S	Cold	6 × 12 × 17	0.64	AE	17 G	20 W	0.18	0.07	0.04	0.02	95.42
19	39/F	M	Cold	40 × 36 × 46	34.68	AE	17 G	25 W	17.53	9.71	6.36	4.6	86.71
20	24/F	M	Cold	27 × 32 × 50	22.61	AE	17 G	20 W	10.77	7.88	5.6	4.02	82.18
21	61/F	S	Cold	35 × 35 × 40	2.565	C	17 G	30 W	15.48	11.57	9.95	7.85	7.87
22	58/F	S	Hot	18 × 17 × 35	5.6	C,AE	17 G	30 W	2.9	2	1.42	1.13	79.83
23	62/F	S	Cold	24 × 44 × 50	27.64	AE	17 G	25 W	6.16	4.8	3.25	2.49	90.98
24	65/F	S	Cold	27 × 29 × 36	14.75	C	18 G	15 W	12.12	9.8	8.67	7.15	51.53
25	54/F	S	Cold	24 × 24 × 47	14.17	C	18 G	15 W	8.23	6.04	5.24	4.48	68.35
26	44/F	S	Cold	30 × 22 × 39	13.47	C	17 G	30 W	7.37	4.47	3.1	2.22	83.49
27	38/F	S	Hot	10 × 15 × 18	1.41	H	18 G	15 W	0.34	0.11	0.025	0.009	99
28	52/F	S	Hot	18 × 19 × 20	3.58	H	18 G	15 W	2.22	1.58	1.19	0.87	75.43
29	49/F	M	Cold	22 × 36 × 39	16.17	AE	17 G	20 W	9.39	7.79	2.63	2.72	88.11
30	48/F	M	Cold	24 × 31 × 42	16.36	AE	17 G	20 W	4.56	3.07	1.67	1.17	92.81

^a^S, solid; E, spongiform; M, mix.

^b^G, growing nodule; H, hyperthyroidism; AE, aesthetic; C, compression.

VRR, volume reduction rate calculated from baseline to the end of the follow-up.

### Demographic data and treatment indications

The mean age of the patients included in the database was 49.5 years, and 96.7% of the patients were women and 23.3% were men.

Treatments were mainly indicated for aesthetic concerns, i.e. single indication in 40.0% of the cases and combined with other indications in 63.3%. In 7 of the patients (23.3%), the main indication for treatment was hyperthyroidism and in 5 of them, it was the only indication. Around 16 of the patients were euthyroid, 2 had hyperthyroidism, 7 had subclinical hyperthyroidism, 3 were hypothyroid, and 2 had subclinical hypothyroidism.

### Characteristics of the nodule to be treated

Nodules had a spongiform composition in 6.7% of the patients, mixed composition in 23.3%, and 70.0% were solid. Around 16 of the nodules were located in the left lobe and 14 in the right lobe. In the color Doppler ultrasound study, peripheral flow was detected in 15 patients, mixed flow in 8 of them, and no flow in 7 of them.

On gammagraphy, 76.7% of the cases were cold and 23.3% were hot. The thyroid function of the patients was normal in 53.3%, hypothyroid in 16.7%, and hyperthyroid in 30.0%. The seven patients with hot nodules on gammagraphy had hyperthyroidism, five of them had subclinical, and two had classic symptomatology (Table 1).

### Thickness and applied power

The electrode used had a thickness of 17 G in 70.0% of the cases and 18 G in the remaining 30.0%. The power applied was 15 W in 30.0% of the patients, 20 W in 30.0%, 25 W in 30.0%, and 30 W in the remaining 10.0% (Table 1).

### Complications

Regarding complications, no cases of major complications were recorded. Only one (3.3%) patient presented minor complications consisting of a transient aphonia. This patient started with aphonia immediately after the procedure and vocal cord motility was evaluated showing paralysis of the ipsilateral one. We started intensive medical treatment with descending corticosteroid therapy, and she was subsequently referred to a speech therapist. At 3 months, she had completely recovered her voice and bilateral mobility of both vocal cords was confirmed.

### Volume reduction

**Figure 3:**
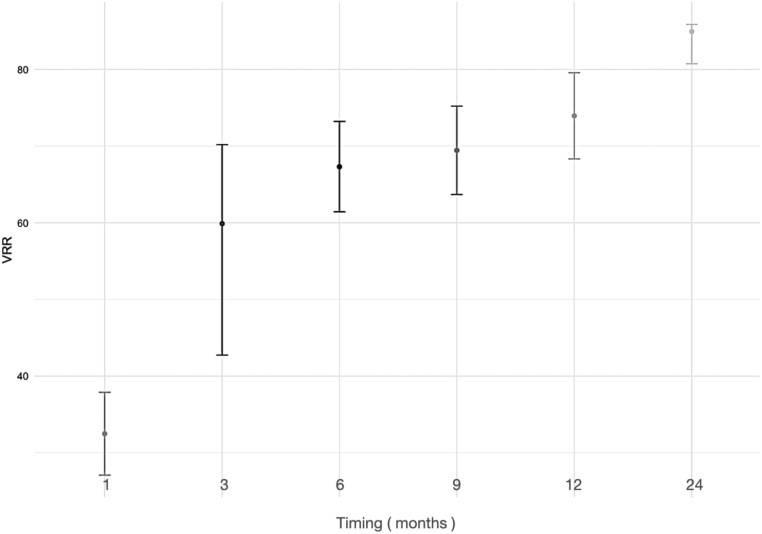
Volume reduction rate over time after microwave ablation in thyroid nodules.

**Figure 4:**
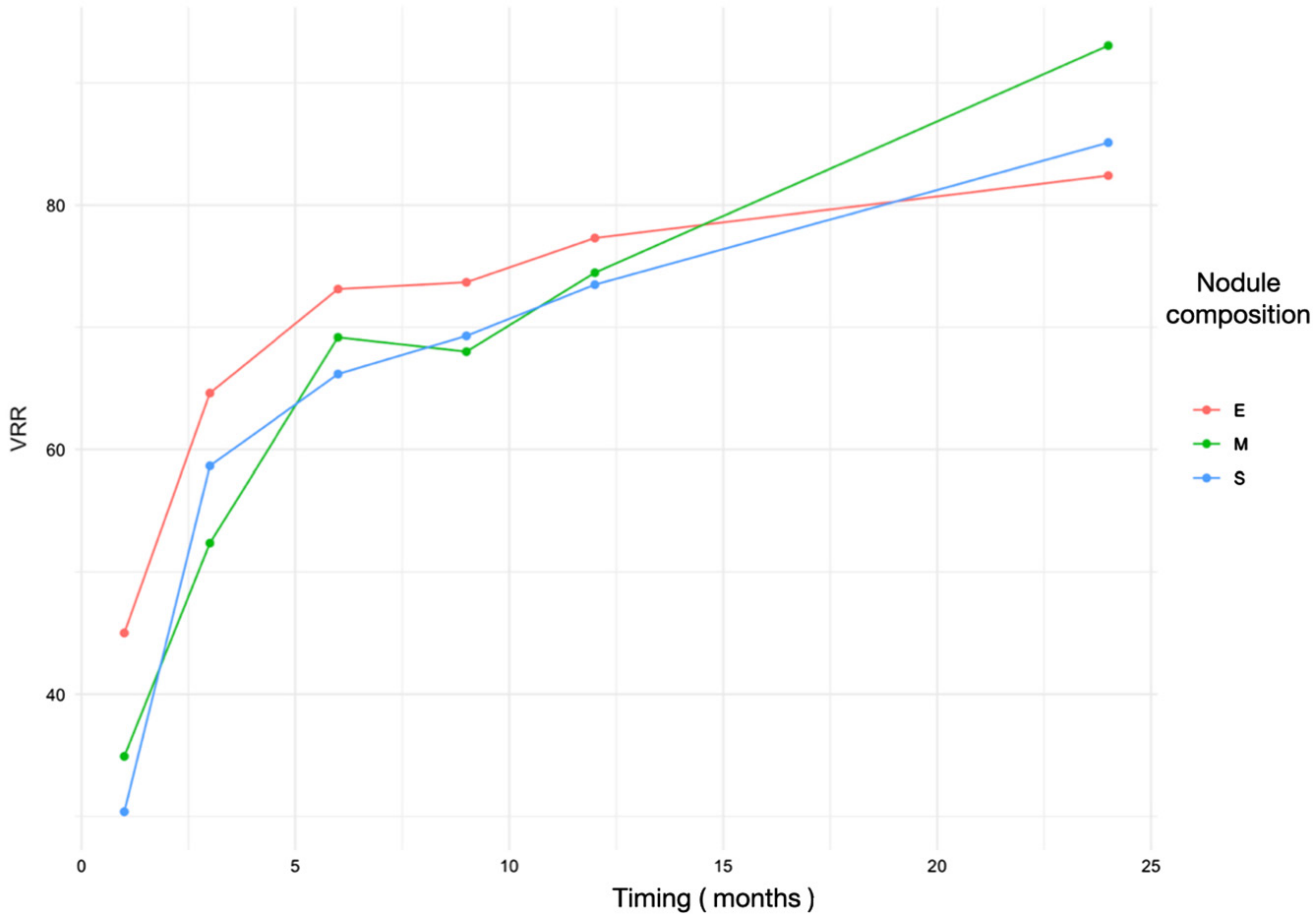
VRR in nodules of different compositions over time showing how all have comparable results and a desirable reduction in volume. E, spongiform; M, mixed; S, solid.

The pre-ablation nodular size and nodular size over time were studied, and the nodular size decreased at 1 month by 32.48%, 3 months by 59.88%, 6 months by 67.31%, 12 months by 73.94%, and 24 months by 81.81% (Fig. 3 and Table 1). A comparative statistical analysis of the VRR was performed according to the composition of the treated nodules, differentiating whether the nodules had a solid, mixed, or spongiform composition (Fig. 4). No significant differences were found.

### Symptom and cosmetic score: overall satisfaction rating

The mean symptom score at the pretreatment time was 4.00, whereas it was 0.00 at the post-treatment. Almost significant differences (*P*  < 0.10) were found between the symptom score means at the time of treatment, and the post-treatment score was lower. This trend could be confirmed in future studies with a larger number of cases.

The mean cosmetic score at the pretreatment time was 3.00, whereas at the post-treatment time, it dropped to 1.00. Significant differences were found (*P*  < 0.05) between the pre- and post-treatment groups, with lower score in the post-treatment group.

The degree of overall satisfaction was average, which presents a median value of 9.00 among the treated patients.

### Evolution in ablated hot nodules

The seven patients who presented hot nodules in the gammagraphy and hyperthyroidism had normalized thyroid function before the first 6 months. In two symptomatic patients, symptoms disappeared during the third-month control. The median TSH value in patients with hot nodules was 0.04 at baseline and 0.72 at the 12-month follow-up. Volume reduction rate was 82.48%, with no significant differences from that found in cold nodules (81.56%) and the total number of ablated nodules (81.81%).

## Discussion

RFA was demonstrated to be an effective and safe alternative to surgery and is recommended in the European guidelines as the first-line TA treatment modalities along with laser ablation (7). In comparative publications between the use of radiofrequency and microwave, a non-significant superiority in the VRR and lower rate of complications were found with the former (12, 13, 14). However, none of them used a microwave antenna specifically designed for TNs, and more importantly, they used, both in RFA and MWA, the MST, which is necessary for the safe and effective use of radiofrequency but not for microwaves.

MWA is a feasible alternative to RFA that could potentially overcome some radiofrequency limitations if a dedicated device and technique are used. The potential advantages of MWA over RFA include the following: MWA allows a larger zonal ablation and does not suffer or does so to a lesser degree, the well-known effects of heat theft caused by adjacent or intratumoral vessels (8). The impedance will be less affected by scar tissue generated by previous ablations being the technique of choice in many centers when we find a remnant or tumor recurrence after ablation (16, 17). Finally, the use of grounding pads will not be necessary, and it can be used in patients with pacemakers.

Up to now, MWA for TN was performed with cooled antennas marketed with quite large diameters, different from the thin antennas that are required for thyroid ablation. Moreover, cooled antennas are bulky and connected to different cables (water and energy); therefore, precise insertion and repositioning could be cumbersome. The novel MWA system that we utilized in our clinical practice is non-cooled, and the thin, lightweight antennas we used (17–18 G, 10–8 cm in length, 20–25 g each) were easily detachable from the energy cable, offering great precision during the procedure.

Baek *et al.* (18) suggested that the fixed electrode technique (FET) ablation with monopolar radiofrequency was not suitable for the thyroid gland, since unlike the liver, the thyroid gland is a small organ, whereas TNs are relatively large and ellipsoid in shape. Therefore, the treatment strategy of FET making a round-shaped ablation zone is dangerous to surrounding critical structures in the thyroid gland. Alternatively, MST (19) consisted of the compartmentalization of the nodule into multiple imaginary or supposed ablating units that are then treated individually. The ablations of each of the compartments have a very short duration (5–10 s) to prevent radiofrequency cutoff, and the antenna must be mobilized very quickly within the nodule to achieve a safe and complete ablation. Therefore, it requires frequent repositioning, exposing the patient to theoretically high risk of bleeding. In addition, in large nodules and theoretically small ablative compartments, the procedures can be very lengthy and even complicated by technical difficulties because of the steam generated.

**Figure 5:**
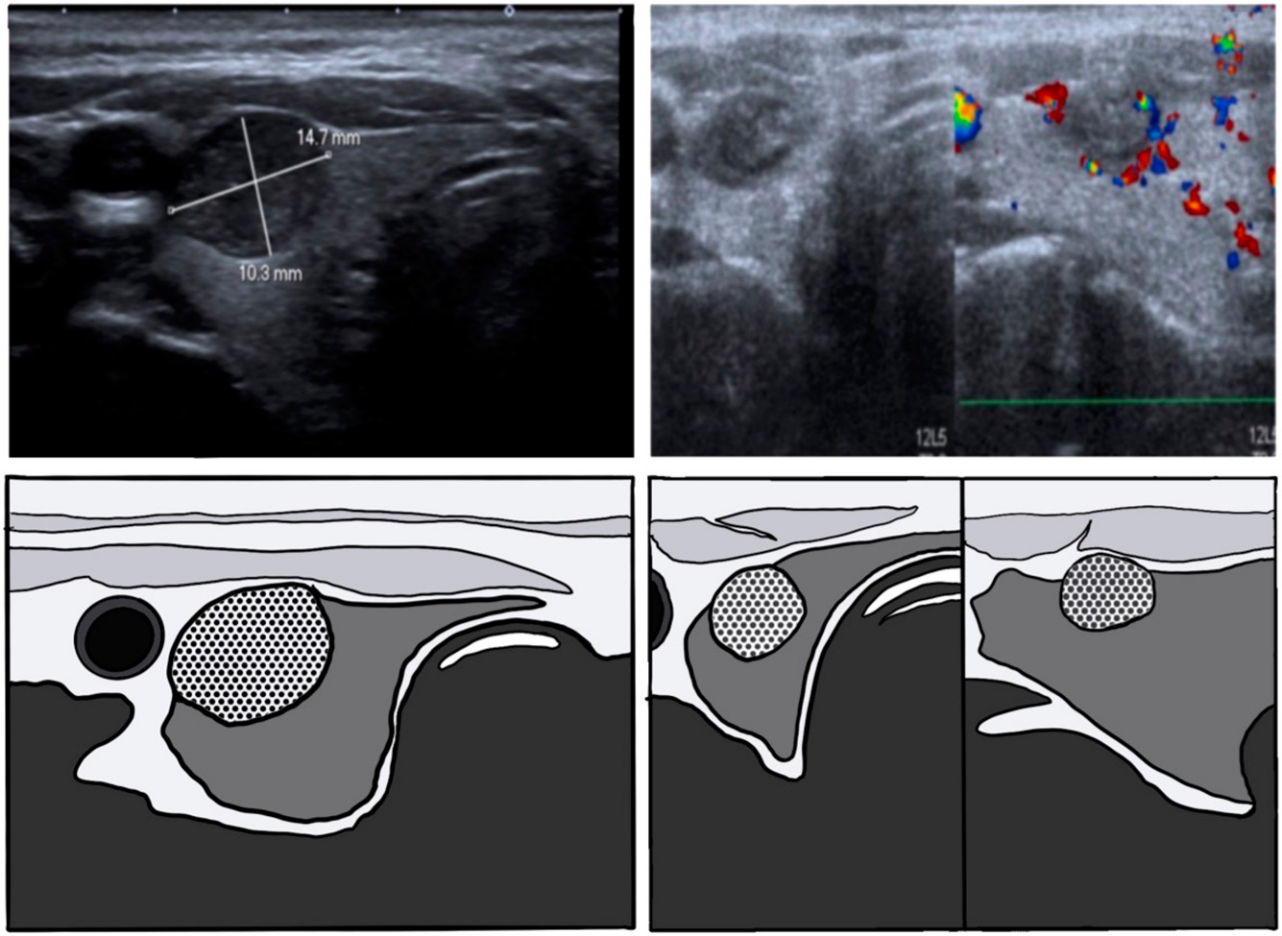
Usual reduction in the size of the nodule and alteration of the echogenicity in the ultrasound control at 12 months after ablation.

The MOA technique is a good alternative in the case of MWA or bipolar RFA for TNs (20). In our experience of treating nodules with a maximum diameter of 2 cm, we only needed four to five overlapping ablations of no more than 2 min for each one to cover >80% of the nodule (Fig. 5). In MWA, we also benefit from the absence of heat theft caused by the presence of cooling vessels inside the nodule and from the difficulties of RFA that we usually find in scar areas or previous ablations. Since the ablation of a nodule is achieved with less antenna repositioning, we should have less bleeding complications, especially when using a dedicated fine MW device.

With the MST, starting the ablation in the deepest and most remote part of the nodule is important because a hyperechogenic area is the only US sign that is obtained with the ablation of each compartment, which will make it impossible to visualize this area later on. Since microwave antennas use lower power, the microbubbles generated are less troublesome than with radiofrequency antennas. Safety is linked to visibility; therefore, continuous visualization of the procedure allows us to obtain precise and controlled ablation zones with margins sharply delimitated with US.

The results we have obtained in terms of safety, volume reduction, and symptom improvement are similar to those found in longer series of radiofrequency treatment of benign thyroid nodules (11, 12, 13, 14).

However, the favorable response in this small number of cases should be confirmed in future studies using the technique described in this article.

## Conclusions

The MOA technique with a dedicated uncooled MW antenna for thyroid ablation was successfully utilized for all our treatments, leading to an important VRR, an acceptable safety profile, great improvement of symptoms, and a very high level of patient satisfaction. Thus, MWA could be a good alternative to surgery and RFA in the management of BTNs.

## Declaration of interest

The authors declare no conflict of interest.

## Funding

This work did not receive any specific grant from any funding agency in the public, commercial or not-for-profit sector.

## Author contribution statement

All authors have substantially contributed to the wok reported and agreed on the final publish version of the manuscript.

## References

[1] Gharib H Papini E Garber JR Duick DS Harrell RM Hegedüs L Paschke R Valcavi R Vitti P AACE/ACE/AME Task Force on Thyroid Nodules, American college of endocrinology, and associazione medici endocrinologi medical guidelines for clinical practice for the diagnosis and management of thyroid nodules - 2016 update appendix. Endocrine Practice 2016 22 622–639. (10.4158/EP161208.GL) 27167915

[2] Hegedüs L Frasoldati A Negro R Papini E. European Thyroid Association survey on use of minimally invasive techniques for thyroid nodules. European Thyroid Journal 2020 9 194–204. (10.1159/000506513) 32903971 PMC7445736

[3] Papini E Monpeyssen H Frasoldati A Hegedüs L. 2020 European Thyroid Association clinical practice guideline for the use of image-guided ablation in benign thyroid nodules. European Thyroid Journal 2020 9 172–185. (10.1159/000508484) 32903999 PMC7445670

[4] Patel KN Yip L Lubitz CC Grubbs EG Miller BS Shen W Angelos P Chen H Doherty GM Fahey TJ 3rd The American Association of Endocrine Surgeons guidelines for the definitive surgical management of thyroid disease in adults. Annals of Surgery 2020 271 e21–e93. (10.1097/SLA.0000000000003580) 32079830

[5] Mathonnet M Cuerq A Tresallet C Thalabard JC Fery-Lemonnier E Russ G Leenhardt L Bigorgne C Tuppin P Millat B What is the care pathway of patients who undergo thyroid surgery in France and its potential pitfalls? A national cohort. BMJ Open 2017 7 e013589. (10.1136/bmjopen-2016-013589) PMC5558818 28389487

[6] Zhi X Zhao N Liu Y Liu JB Teng C Qian L. Microwave ablation compared to thyroidectomy to treat benign thyroid nodules. International Journal of Hyperthermia 2018 34 644–652. (10.1080/02656736.2018.1456677) 29577796

[7] Bernardi S Dobrinja C Fabris B Bazzocchi G Sabato N Ulcigrai V Giacca M Barro E De Manzini N Stacul F. Radiofrequency ablation compared to surgery for the treatment of benign thyroid nodules. International Journal of Endocrinology 2014 2014 934595. (10.1155/2014/934595) PMC4090443 25045352

[8] Cheng Z Liang P. Advances in ultrasound-guided thermal ablation for symptomatic benign thyroid nodules. Advances in Clinical and Experimental Medicine 2020 29 1123–1129. (10.17219/acem/125433) 32926600

[9] Shi YF Zhou P Zhao YF Liu WG Tian SM Liang YP. Microwave ablation compared with laser ablation for treating benign thyroid nodules in a propensity-score matching study. Frontiers in Endocrinology (Lausanne) 2019 10 874. (10.3389/fendo.2019.00874) PMC6923173 31920983

[10] Mainini AP Monaco C Pescatori LC De Angelis C Sardanelli F Sconfienza LM Mauri G. Image-guided thermal ablation of benign thyroid nodules. Journal of Ultrasound 2017 20 11–22. (10.1007/s40477-016-0221-6) 28298940 PMC5334266

[11] Cheng Z Che Y Yu S Wang S Teng D Xu H Li J Sun D Han Z Liang P. US-guided percutaneous radiofrequency versus microwave ablation for benign thyroid nodules: a prospective multicenter study. Scientific Reports 2017 7 9554. (10.1038/s41598-017-09930-7) PMC5573330 28842651

[12] Yue WW Wang SR Lu F Sun LP Guo LH Zhang YL Li XL Xu HX. Radiofrequency ablation vs. microwave ablation for patients with benign thyroid nodules: a propensity score matching study. Endocrine 2017 55 485–495. (10.1007/s12020-016-1173-5) 27905049

[13] Hu K Wu J Dong Y Yan Z Lu Z Liu L. Comparison between ultrasound-guided percutaneous radiofrequency and microwave ablation in benign thyroid nodules. Journal of Cancer Research and Therapeutics 2019 15 1535–1540. (10.4103/jcrt.JCRT_322_19) 31939434

[14] Cervelli R Mazzeo S De Napoli L Boccuzzi A Pontillo-Contillo B Materazzi G Miccoli P Cioni R Caramella D. Radiofrequency ablation in the treatment of benign thyroid nodules: an efficient and safe alternative to surgery. Journal of Vascular and Interventional Radiology 2017 28 1400–1408. (10.1016/j.jvir.2017.07.009) 28844832

[15] Wright AS Sampson LA Warner TF Mahvi DM Lee FT Jr. Radiofrequency versus microwave ablation in a hepatic porcine model. Radiology 2005 236 132–139. (10.1148/radiol.2361031249) 15987969

[16] Brace CL Radiofrequency and microwave ablation of the liver, lung, kidney, and bone: what are the differences? Current Problems in Diagnostic Radiology 2009 38 135–143. (10.1067/j.cpradiol.2007.10.001) 19298912 PMC2941203

[17] Ha EJ Baek JH Lee JH. Moving-shot versus fixed electrode techniques for radiofrequency ablation: comparison in an ex-vivo bovine liver tissue model. Korean Journal of Radiology 2014 15 836–843. (10.3348/kjr.2014.15.6.836) 25469097 PMC4248641

[18] Jeong WK Baek JH Rhim H Kim YS Kwak MS Jeong HJ Lee D. Radiofrequency ablation of benign thyroid nodules: safety and imaging follow-up in 236 patients. European Radiology 2008 18 1244–1250. (10.1007/s00330-008-0880-6) 18286289

[19] Kohlhase KD Korkusuz Y Gröner D Erbelding C Happel C Luboldt W Grünwald F. Bipolar radiofrequency ablation of benign thyroid nodules using a multiple overlapping shot technique in a 3-month follow-up. International Journal of Hyperthermia 2016 32 511–516. (10.3109/02656736.2016.1149234) 27126512

[20] Klebe J Happel C Grünwald F Korkusuz H. Visualization of tissue alterations in thyroid nodules after microwave ablation: sonographic versus scintigraphic imaging. Nuclear Medicine Communications 2015 36 260–267. (10.1097/MNM.0000000000000242) 25369752

